# *BRAF* ^AMP^ Frequently Co-occurs With *IDH1/2*, *TP53*, and *ATRX* Mutations in Adult Patients With Gliomas and Is Associated With Poorer Survival Than That of Patients Harboring *BRAF* ^V600E^

**DOI:** 10.3389/fonc.2020.531968

**Published:** 2021-01-07

**Authors:** Rong Da, Maode Wang, Haitao Jiang, Tuo Wang, Wei Wang

**Affiliations:** ^1^ Department of Clinical Laboratory, The First Affiliated Hospital of Xi’an Jiaotong University, Xi’an, China; ^2^ Department of Neurosurgery, The First Affiliated Hospital of Xi’an Jiaotong University, Xi’an, China

**Keywords:** *BRAF*, *IDH1*/2, *TP53*, *ATRX*, glioma, copy number amplification, mutation, overall survival

## Abstract

Abnormal RAS/RAF signaling plays a critical role in glioma. Although it is known that the V600E mutation of v-raf murine viral oncogene homolog B1 (*BRAF* ^V600E^) and *BRAF* amplification (*BRAF* ^AMP^) both result in constitutive activation of the RAS/RAF pathway, whether *BRAF* ^V600E^ and *BRAF* ^AMP^ have different effects on the survival of glioma patients needs to be clarified. Using cBioPortal, we retrieved studies of both mutations and copy number variations of the *BRAF* gene in CNS/brain tumors and investigated data from 69 nonredundant glioma patients. The *BRAF* mutation group had significantly more male patients (64.00% vs. 36.84%; *P* = 0.046) and a higher occurrence of glioblastoma multiforme (66.00% vs. 31.58%; *P* = 0.013) compared to those in the other group. The *BRAF* ^AMP^ group had significantly more patients with the mutant isocitrate dehydrogenase 1 and 2 (*IDH1/2*) (73.68% vs. 18.00%; *P* = 0.000), tumor protein p53 (*TP53*) (73.68% vs. 30.00%; *P* = 0.002), and alpha thalassemia/mental retardation syndrome X linked (*ATRX*) (63.16% vs. 18.00%; *P* = 0.001) than the mutation group. The *BRAF* ^AMP^ and *IDH1/2* ^WT^ cohort had lower overall survival compared with the *BRAF* ^AMP^ and *IDH1/2* ^MT^ groups (*P* = 0.001) and the *BRAF* mutation cohort (*P* = 0.019), including the *BRAF* ^V600E^ (*P* = 0.033) and *BRAF* ^non-V600E^ (*P* = 0.029) groups, using Kaplan–Meier survival curves and the log rank (Mantel–Cox) test. The *BRAF* ^AMP^ and *IDH1/2* ^WT^ genotype was found to be an independent predictive factor for glioma with *BRAF* mutation and *BRAF* ^AMP^ using *Cox* proportional hazard regression analysis (HR = 0.138, *P* = 0.018). Our findings indicate that *BRAF* ^AMP^ frequently occurs with *IDH1/2*, *TP53*, and *ATRX* mutations. Adult patients with glioma with *BRAF* ^AMP^ and *IDH1/2* ^WT^ had worse prognoses compared with those with *BRAF* mutation and *BRAF* ^AMP^ and *IDH1/2* ^MT^. This suggests that the assessment of the status of *BRAF* ^AMP^ and *IDH1/2* in adult glioma/glioblastoma patients has prognostic value as these patients have relatively short survival times and may benefit from personalized targeted therapy using *BRAF* and/or MEK inhibitors.

## Introduction

Gliomas are the most frequent primary brain neoplasms occurring in both the pediatric and adult populations (1). The 2016 WHO Classification of Tumors of the Central Nervous System was the first to provide combined data regarding the genetic and histological characteristics of tumors and is, thus, considered a cornerstone for understanding and diagnosing tumors. When diagnosing the disease, mutation site genotypes of genes such as isocitrate dehydrogenase *(IDH)*, tumor protein p53(*TP53*), and alpha thalassemia/mental retardation syndrome X linked *(ATRX)* and 1p/19q codeletion should be evaluated. Hence, determining the status of *IDH* mutation and 1p/19q is essential for the 2016 classification of diffused gliomas, including astrocytoma, oligoastrocytoma, oligodendroglioma, and glioblastoma (2). The RAS/RAF/MEK/extracellular signal-regulated kinase (ERK) mitogen-activated protein kinase (MAPK) pathway, which transduces mitogenic stimuli *via* the activation of growth factor receptors, is critical for cell proliferation, survival, and differentiation. Abnormal activation of RAS/RAF signaling plays a role in various tumors, and studies have revealed that the MAPK pathway is of great clinical significance in gliomas (3). Oncogenic mutations as well as the copy number amplification of RAS/RAF and/or abnormal activation of upstream growth factor receptors can cause hyperactivation of the RAS/RAF pathway (4), resulting in various neoplasms.

*BRAF* (v-raf murine viral oncogene homolog B1) participates in the pathological mechanism of 7% of human neoplasms, especially in melanoma, colorectal, thyroid, and lung cancers (5, 6). Because of the negative outcome of high-grade glioma, *BRAF* mutations have gained considerable interest in the possible benefit of the MAPK pathway inhibitors for glioma treatment. The *BRAF* ^V600E^ mutation in which the thymine at nucleotide 1799 is substituted by adenine results in the substitution of valine with glutamic acid at amino acid 600; this is the most common *BRAF* mutation in glioma (6). It imitates the normal phosphorylation of T599 and S602, resulting in the overactivation of *BRAF* kinase and hyperactivation of the ERK signaling pathway (7). In addition, *BRAF* amplification (*BRAF* ^AMP^) can also cause hyperactivation of MAPK signaling, which plays essential roles in the acquired resistance to MAPK inhibitor therapy in cancers harboring *BRAF* ^V600E^ (8). Moreover, *BRAF* ^AMP^ is also found in primary pediatric low-grade gliomas (9).

Although both *BRAF* ^V600E^ and *BRAF* ^AMP^ can lead to the hyperactivation of MAPK signaling, the differences between the patterns of *BRAF* ^V600E^ and *BRAF* ^AMP^ signaling in glioma, their influence on the survival of glioma patients, and the involvement of other genes, remains unclear. In this study, based on cBioPortal data, we found that patients with glioma harboring *BRAF* ^AMP^ had lower overall survival compared with those harboring *BRAF* ^V600E^. Furthermore, we found that *BRAF* ^AMP^ frequently co-occurred with *IDH1/2*, *TP53*, and *ATRX* mutations.

## Materials and Methods

### Data Collection and Enrollment

We used cBioPortal (https://www.cbioportal.org/) (10, 11). The Cancer Genome Atlas Program (TCGA) data mining tool to collect the necessary data. TCGA is a public database, and we strictly followed its publication guidelines (https://www.cancer.gov/about-nci/organization/ccg/research/structural-genomics/tcga/using-tcga/citing-tcga) for collecting and generating data. Multiple patient cohorts, including all 19 available studies on central nervous system (CNS)/brain tumors (6122 samples) were queried. The data were filtered to include studies that listed both gene mutation and copy number data. In each study, mutations and putative copy number alterations (CNA) identified using the Genomic Identification of Significant Targets in Cancer (GISTIC) tool were selected to analyze the genomic profiles. We first selected tumor samples with mutations and CNA data for creating the patient/case set. Then, the gene names *BRAF*, *ATRX*, *TP53*, *IDH1*, and *IDH2* were entered, and the query was submitted. Among the retrieved data files, we selected samples harboring the *BRAF* mutation with AMP. The mutation data and CNA as well as the patient and sample data were retrieved. All data were recorded in a chart for further analysis.

### Characteristics Associated With *BRAF* ^AMP^ and *BRAF* Mutation in Glioma Using Univariate and Multivariate Logistic Regression Analysis

The study population was divided into the *BRAF* ^AMP^ and *BRAF* mutation groups, and the numerical values of the categorical variables were calculated. The demographic characteristics of the patients, pathological classification, and molecular biomarkers in the two groups were analyzed using univariate logistic regression analysis. Then, the statistically significant variables (*P* < 0.10) were analyzed using multivariate logistic regression analysis. The odds ratios and 95% confidence intervals were estimated. *P* value < 0.05 was considered statistically significant. For greater precision of characteristic evaluation, we created a descriptive table and divided the *BRAF* ^AMP^ group into two groups based on the non- and co-occurrence of the *IDH1/2* mutation, and the *BRAF* mutation group into *BRAF* ^V600E^ and *BRAF* ^non-V600E^ groups.

### Cross-Over Analysis Using Kaplan–Meier Survival Curves and the Log Rank (Mantel–Cox) Test

The overall survival of the *BRAF* ^AMP^ and *IDH1/2* ^MT^, *BRAF* ^AMP^ and *IDH1/2* ^WT^, *BRAF* ^V600E^, and *BRAF* ^non-V600E^ groups was determined by a crossover comparison using Kaplan–Meier survival curves and the log rank (Mantel–Cox) test (12). The survival of the *BRAF* mutation group was compared with that of the *BRAF* ^AMP^ and *IDH1/2* ^MT^ and *BRAF* ^AMP^ and *IDH1/2* ^WT^ groups, respectively. *P* value < 0.05 was considered statistically significant.

### Multivariate Analysis of Overall Survival Using Cox Regression Analysis

The *BRAF* ^AMP^ and *IDH1/2* ^WT^, *TP53*, and *ATRX* were analyzed using the Cox regression analysis in the 69 samples with *BRAF* ^AMP^ or *BRAF* mutation. *P* value < 0.05 was considered statistically significant.

### String Analysis of BRAF, IDH1, IDH2, TP53, and ATRX

Using STRING: functional protein association networks (https://string-db.org/) (13), the association among BRAF, IDH1, IDH2, TP53, and ATRX was investigated, and the combined scores among those four proteins were obtained.

## Results

### Data Enrollment in the Study

**Figure 1:**
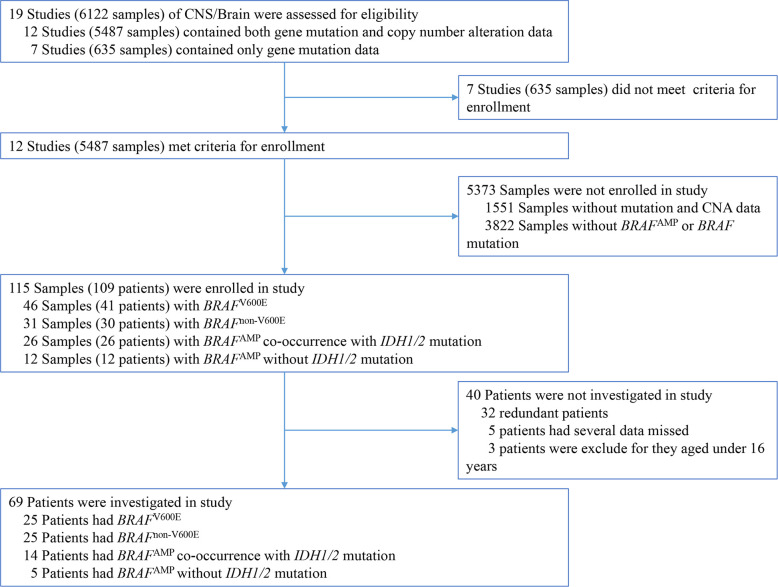
Schematic representation of the process of data enrollment in the study using cBioPortal. Among the 19 CNS/brain studies, including 6112 samples, 115 samples (109 patients) with *BRAF* mutation or *BRAF* ^AMP^ were enrolled in this study, and 69 nonredundant patients with information regarding major patient characteristics, including sex, age, cancer type details, *BRAF* mutation, *BRAF* copy number alteration, and mutation of *IDH1/2*, *TP53*, as well as *ATRX*, were further investigated. BRAF, v-raf murine viral oncogene homolog B1; IDH1/2, isocitrate dehydrogenase 1 and 2.

Among the 19 CNS/brain studies available (6122 samples), 12 studies (5487 samples) matched the required inclusion criteria, containing both gene mutation and CNA data (**Table 1**). The cancer types in these 12 CNS/brain studies included diffuse glioma, glioblastoma, oligodendroglioma, and miscellaneous neuroepithelial tumors. A schematic representation of the flow of data screening and enrollment is shown in **Figure 1**. A total of 115 samples (109 patients) with *BRAF* mutation or *BRAF* ^AMP^ were enrolled in this study, and data from 69 nonredundant patients were investigated. Integrated data of major patient characteristics, including sex, age, cancer type, *BRAF* mutation, *BRAF* CNA, and mutation of *IDH1/2*, *TP53*, and *ATRX*, were collected for further analysis (**Supplementary Table S1**).

**Table 1:** The CNS/Brain projects of TCGA data enrolled in the study retrieved using cBioPortal.

Project	All Samples	Samples with mutation and CNA data	Samples of *BRAF* ^AMP^	Samples of *BRAF* ^V600E^	Samples of *BRAF* ^non-V600E^	References
Diffuse Glioma						
Brain Lower Grade Glioma (TCGA, Firehose Legacy)	530	283	10	1	1	https://www.cancer.gov
Brain Lower Grade Glioma (TCGA, PanCancer Atlas)	514	507	7	1	2	(14–19)
Glioma (MSK, 2018)	91	91	1	2	1	https://www.cancer.gov
Glioma (MSKCC, Clin Cancer Res 2019)	1004	1004	3	22	26	(20)
Merged Cohort of LGG and GBM (TCGA, Cell 2016)	1102	794	9	5	2	(21)
Glioblastoma						
Brain Tumor PDXs (Mayo Clinic, 2019)	95	83	0	2	1	https://www.cbioportal.org
Glioblastoma (TCGA, Cell 2013)	543	248	2	3	0	(22)
Glioblastoma (TCGA, Nature 2008)	206	91	0	0	0	(23)
Glioblastoma Multiforme (TCGA, Firehose Legacy)	604	273	1	5	1	https://www.cancer.gov
Glioblastoma Multiforme (TCGA, PanCancer Atlas)	592	378	4	5	4	(14–19, 24)
Oligodendroglioma						
Anaplastic Oligodendroglioma and Anaplastic Oligogastrocytoma (MSKCC, Neuro Oncol 2017)	22	22	0	0	0	(25)
Miscellaneous Neuroepithelial Tumor						
Pheochromocytoma and Paraganglioma (TCGA, Firehose Legacy)	184	162	1	0	1	https://www.cancer.gov

### Characteristics Associated With *BRAF* ^AMP^ and *BRAF* Mutation of Glioma

The study population was divided into two groups, *BRAF* ^AMP^ and *BRAF* mutation. The demographic characteristics and clinical data of the two groups are summarized in **Table 2**. The age of patients ranged from 20 to 85 years with an average of 45.46 years. Twenty-five patients harbored *BRAF* ^non-V600E^ mutations; of these, two patients harbored a D594G mutation; two patients, a G469A mutation; and the remaining patients, an A320T mutation combined with A171E, A404Cfs*9, E375*, G466E, G466V, G469R, G469V, G596D, G69S, L331F, L382V, L597R, M531, P708S, S394P, S614P, T121I, V504_R506dup, V504I, W476*, and X709_splice mutations. The *BRAF* mutation group had significantly more male patients (64.00% vs. 36.84%; *P* = 0.046) and a higher occurrence of glioblastoma multiforme (66.00% vs. 31.58%; *P* = 0.013). In contrast, the *BRAF* ^AMP^ group had significantly more patients harboring *IDH1/2* (73.68% vs. 18.00%; *P* = 0.000), *TP53* (73.68% vs. 30.00%; *P* = 0.002), and *ATRX* (63.16% vs. 18.00%; *P* = 0.001) mutations. Variables with *P* value < 0.10 were analyzed using multivariate logistic regression analysis; the *BRAF* mutation group had more male patients (64.00% vs. 36.84%; *P* = 0.027), and the *BRAF* ^AMP^ group had significantly more patients harboring *IDH1/2* mutations (73.68% vs. 18.00%; *P* = 0.029) (**Table 2**). Further analysis indicated that the *BRAF* ^AMP^ group had no simultaneously detected *BRAF* mutations, and that the *BRAF* mutation group had no simultaneously detected *BRAF* ^AMP^. The *BRAF* ^AMP^ and *IDH1/2* ^MT^ group had a significantly higher percentage of co-occurrence of *TP53* (13/14, 92.86%) and *ATRX* (12/14, 85.71%) mutations (**Table 3**).

**Table 2:** Univariate and multivariate analysis: characteristics associated with *BRAF* ^AMP^ and *BRAF* mutation in gliomas.

Variables	*BRAF* ^AMP^(n = 19)		*BRAF* mutation(n = 50)		Univariate analysis			Multivariate analysis		
	Number	%	Number	%	Odds Ratio	95% Confidence Interval	*P* Value	Odds Ratio	95% Confidence Interval	*P* Value
Male	7	36.84	32	64.00	0.328	0.110–0.982	0.046	0.181	0.040–0.824	0.027
Diagnosis Age										
20–40 years	9	47.37	21	42.00	1.243	0.430–2.592	0.688			
41–60 years	7	36.84	18	36.00	1.037	0.346–3.105	0.948			
> 61 years	3	15.79	11	22.00	0.665	0.163–2.704	0.568			
Cancer type detailed										
Glioblastoma multiform	6	31.58	33	66.00	0.238	0.077–0.736	0.013	0.590	0.120–2.893	0.515
Astrocytoma	5	26.32	9	18.00	1.627	0.466–5.680	0.445			
Oligoastrocytoma	4	21.05	0	0.00	5384916143	0.000–	0.999			
Oligodendroglioma	4	21.05	3	6.00	4.178	0.839–20.814	0.081	0.807	0.098–6.633	0.842
Gliosarcoma	0	0.00	2	4.00	0.000	0.000–	0.999			
Other glioma	0	0.00	3	6.00	0.000	0.000–	0.999			
Mutation										
*BRAF* ^V600E^	0	0.00	25	50.00	1227760777	0.000–	0.998			
*BRAF* ^non-V600E^	0	0.00	25	50.00	0.000	0.000–	0.998			
*IDH1/2*	14	73.68	9	18.00	12.756	3.653–44.534	0.000	8.805	1.242–62.406	0.029
*TP53*	14	73.68	15	30.00	6.533	1.994–21.407	0.002	1.463	0.165–13.000	0.733
*ATRX*	12	63.16	9	18.00	7.810	2.403–25.383	0.001	1.832	0.273–12.310	0.534
Copy number variation										
*BRAF* ^AMP^	19	100.00	0	0.00	–	–	–			
Overall survival status										
Deceased	7	36.84	24	48.00	0.632	0.214–1.870	0.407			

**Table 3:** Characteristics associated with *BRAF* ^AMP^ and *BRAF* mutation in gliomas.

Variables	*BRAF* ^AMP^ & *IDH1/2* ^MT^(n = 14)		*BRAF* ^AMP^ & *IDH1/2* ^WT^(n = 5)		*BRAF* ^V600E^(n = 25)		*BRAF* ^non-V600E^(n = 25)	
	Number	%	Number	%	Number	%	Number	%
Male	6	42.86	1	20.00	14	56.00	18	72.00
Diagnosis Age								
20–40 years	7	50.00	2	40.00	12	48.00	9	36.00
41–60 years	6	42.86	1	20.00	7	28.00	11	44.00
> 61 years	1	7.14	2	40.00	6	24.00	5	20.00
Cancer type detailed								
Glioblastoma multiform	2	14.29	4	80.00	19	76.00	14	56.00
Astrocytoma	4	28.57	1	20.00	3	12.00	6	24.00
Oligoastrocytoma	4	28.57	0	0.00	0	0.00	0	0.00
Oligodendroglioma	4	28.57	0	0.00	0	0.00	3	12.00
Gliosarcoma	0	0.00	0	0.00	0	0.00	2	8.00
Other glioma	0	0.00	0	0.00	3	12.00	0	0.00
Mutation								
*BRAF* ^V600E^	0	0.00	0	0.00	25	100.00	0	0.00
*BRAF* ^non-V600E^	0	0.00	0	0.00	0	0.00	25	100.00
*IDH1/2*	14	100.00	0	0.00	0	0.00	9	36.00
*TP53*	13	92.86	1	20.00	1	4.00	14	56.00
*ATRX*	12	85.71	0	0.00	1	4.00	8	32.00
Copy number variation								
*BRAF* ^AMP^	14	100.00	5	100.00	0	0.00	0	0.00
Overall survival status								
Deceased	5	35.71	2	40.00	13	52.00	11	44.00

### Crossover Analysis Using Kaplan–Meier Survival Curves and Log Rank (Mantel–Cox) Test

**Figure 2:**
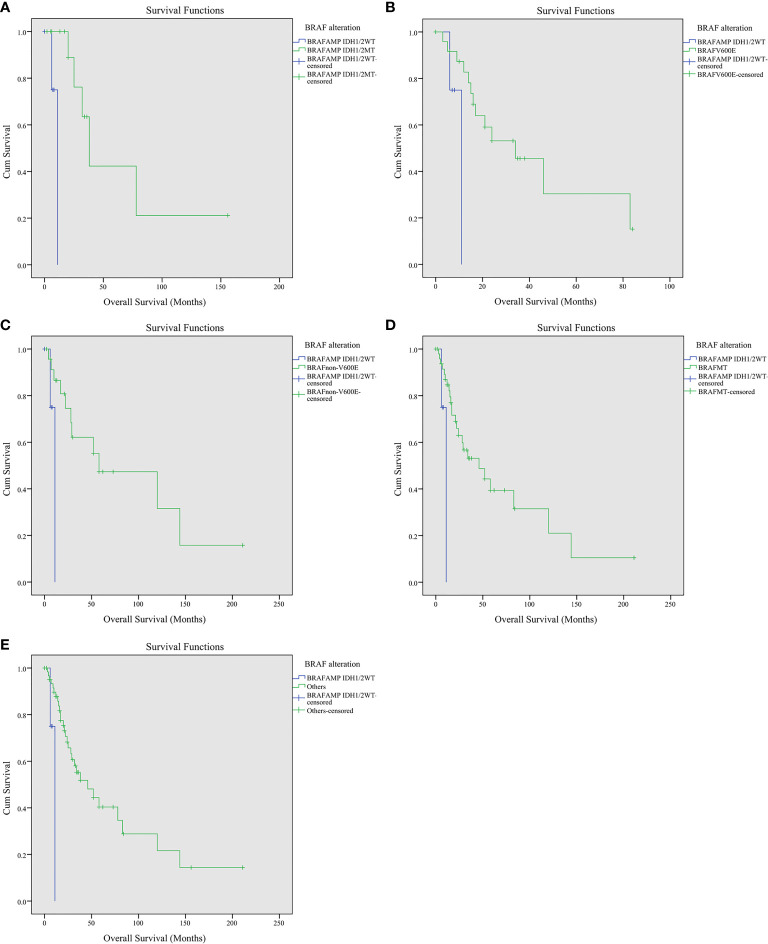
Kaplan–Meier survival curves of patients with gliomas harboring *BRAF* ^AMP^ and *BRAF* mutation. **(A)** *BRAF* ^AMP^ and *IDH1/2* ^WT^ cohort vs. *BRAF* ^AMP^ and *IDH1/2* ^MT^ cohort (9.750 vs. 67.026, chi-square 10.526, *P* = 0.001). **(B)** *BRAF* ^AMP^ and *IDH1/2* ^WT^ cohort vs. *BRAF* ^V600E^ cohort (9.750 vs. 41.573, chi-square 4.536, *P* = 0.033). **(C)** *BRAF* ^AMP^ and *IDH1/2* ^WT^ cohort vs. *BRAF* ^non-V600E^ cohort (9.750 vs. 89.958, chi-square 4.747, *P* = 0.029). **(D)** *BRAF* ^AMP^ and *IDH1/2* ^WT^ cohort vs. *BRAF* mutation cohort (9.750 vs. 71.698, chi-square 5.469, *P* = 0.019). **(E)** *BRAF* ^AMP^ and *IDH1/2* ^WT^ cohort vs. other three *BRAF* alteration cohorts, including the *BRAF* ^AMP^ and *IDH1/2* ^MT^, *BRAF* ^V600E^, and *BRAF* ^non-V600E^ cohorts (9.750 vs. 74.401, chi-square 5.469, *P* = 0.019). BRAF, v-raf murine viral oncogene homolog B1; IDH1/2, isocitrate dehydrogenase 1 and 2.

The crossover Kaplan–Meier survival curves and log rank (Mantel–Cox) test were performed to explore the influence of *BRAF* gene alteration on the overall survival of glioma patients. The estimated mean survival time was 67.026 months for patients harboring *BRAF* ^AMP^ and *IDH1/2* ^MT^, 9.750 months for patients harboring *BRAF* ^AMP^ and *IDH1/2* ^WT^, 41.573 months for patients harboring *BRAF* ^V600E^, and 89.958 months for patients harboring *BRAF* ^non-V600E^. The estimated survival time of the *BRAF* ^AMP^ and *IDH1/2* ^WT^ cohort was the shortest and was significantly lower compared with that of the *BRAF* ^AMP^ and *IDH1/2* ^MT^ (9.750 vs. 67.026, chi-square 10.526, *P* = 0.001), the *BRAF* ^V600E^ (9.750 vs. 41.573, chi-square 4.536, *P* = 0.033), and the *BRAF* ^non-V600E^ (9.750 vs. 89.958, chi-square 4.747, *P* = 0.029) cohorts. The estimated mean survival time of the *BRAF* mutation cohort was significantly greater than that of the *BRAF* ^AMP^ and *IDH1/2* ^WT^ cohort (71.698 vs. 9.750, chi-square 5.469, *P* = 0.019). The estimated mean survival times of the three cohorts were significantly greater than that of the *BRAF* ^AMP^ and *IDH1/2* ^WT^ cohort (74.401 vs. 9.750, chi-square 7.639, *P* = 0.006) (**Figure 2**). When analyzed using Kaplan–Meier survival curves and the log rank (Mantel–Cox) test, there was no significance between the following groups: *BRAF* ^AMP^ cohort vs. *BRAF* mutation cohort (58.835 vs. 71.698, chi-square 0.020, *P* = 0.886), *BRAF* ^V600E^ cohort vs. *BRAF* ^non-V600E^ cohort (41.573 vs. 89.958, chi-square 1.999, *P* = 0.157), *BRAF* ^AMP^ and *IDH1/2* ^MT^ cohort vs. *BRAF* ^V600E^ cohort (67.026 vs. 41.573, chi-square 1.031, *P* = 0.310), *BRAF* ^AMP^ and *IDH1/2* ^MT^ cohort vs. *BRAF* ^non-V600E^ cohort (67.026 vs. 89.958, chi-square 0.025, *P* = 0.875), *BRAF* ^AMP^ and *IDH1/2* ^MT^ cohort vs. *BRAF* mutation cohort (67.026 vs. 71.698, chi-square 0.513, *P* = 0.474) (**Supplementary Figure S1**). The estimated survival time of the *BRAF* ^V600E^ cohort above 30 years of age was 40.135 months, whereas that of the *BRAF* ^AMP^ and *IDH1/2* ^WT^ cohort was significantly lower (9.750 vs. 40.135, chi-square 5.575, *P* = 0.018) (**Supplementary Figure S2**).

### Multivariate Analysis of Overall Survival Using the Cox Regression Analysis

**Figure 3:**
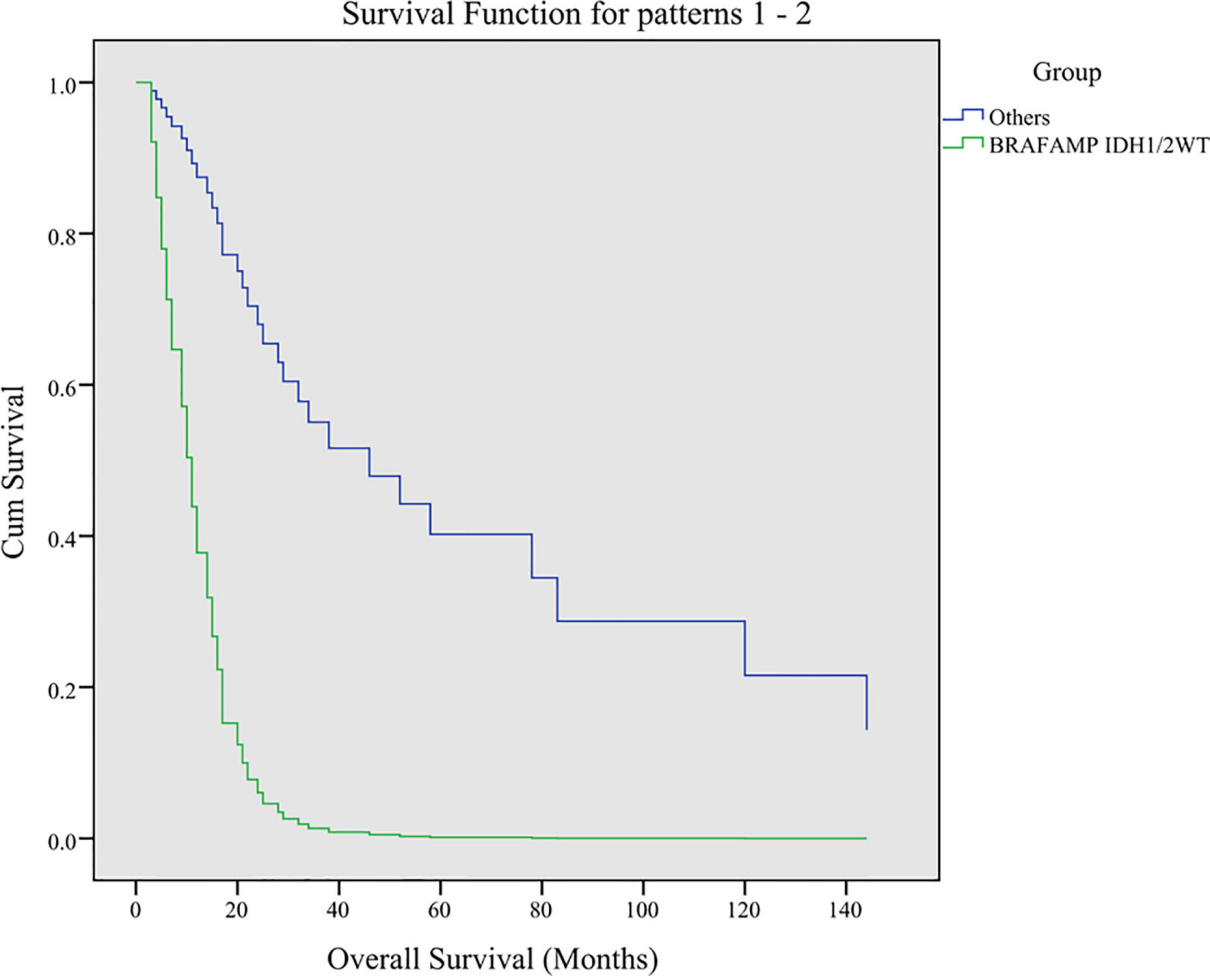
Multivariate analysis of overall survival using Cox regression analysis. Three factors including *BRAF* ^AMP^ and *IDH1/2* ^WT^, *TP53*, and *ATRX* were analyzed. The *BRAF* ^AMP^ and *IDH1/2* ^WT^ genotype was determined as an independent predictive factor for overall survival (HR = 0.138, *P* = 0.018). BRAF, v-raf murine viral oncogene homolog B1; IDH1/2, isocitrate dehydrogenase 1 and 2.

The *IDH1/2* mutation in 13 of the 14 *BRAF* ^AMP^ patients was R132H, and one patient harbored the R132G mutation. The *IDH1/2* mutation in eight *BRAF* ^non-V600E^ patients was R132H with the exception of one sample (R132S). The *TP53* and *ATRX* mutations were highly diverse in all patients (**Supplementary Table S1**). The Cox regression analysis introduced three factors, including *BRAF* ^AMP^ and *IDH1/2* ^WT^, *TP53* mutation, and *ATRX* mutation in all *BRAF* ^AMP^ and *BRAF* mutation patients and determined the *BRAF^AMP^* and *IDH1/2* ^WT^ genotype as an independent predictive factor for overall survival (HR = 0.138, *P* = 0.018) (**Figure 3**).

### Associations Between BRAF, IDH1, IDH2, TP53, and ATRX Using String Analysis

**Figure 4:**
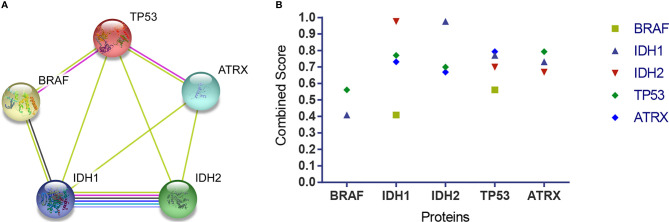
The association among BRAF, IDH1, IDH2, TP53, and ATRX proteins. **(A)** The networks showed functional links among these four proteins, except for BRAF and ATRX and BRAF and IDH2. TP53 is associated with BRAF and ATRX *via* a known interaction (experimentally determined, the pink edge) and another interaction (text mining, the lime green edge), respectively. BRAF is associated with IDH1 *via* other interactions (coexpression, the black edge; text mining, the lime green edge). IDH1 is associated with IDH2 *via* known interactions (from curated databases, the jungle green edge; experimentally determined, the pink edge), predicted interaction (gene co-occurrence, the blue edge), and other interactions (text mining, the lime green edge; coexpression, the black edge; protein homology, the violet edge). TP53 is associated with IDH1 and IDH2, and ATRX is associated with IDH1 and IDH2 *via* another interaction (text mining, the lime green edge) respectively. **(B)** The combined score showed that the highest score was that between IDH1 and IDH2 (0.976), followed by TP53 and ATRX (0.793), TP53 and IDH1(0.770), IDH1 and ATRX (0.731), IDH2 and TP53 (0.700), ATRX and IDH2 (0.669), BRAF and TP53 (0.561), and BRAF and IDH1(0.409). BRAF, v-raf murine viral oncogene homolog B1; IDH1/2, isocitrate dehydrogenase 1 and 2; TP53, tumor protein p53; ATRX, alpha thalassemia/mental retardation syndrome X linked.

The networks showed that there were functional links between BRAF, IDH1, IDH2, TP53, and ATRX except for BRAF and ATRX and BRAF and IDH2. BRAF was directly associated with TP53 and indirectly interacted with ATRX through TP53. BRAF was directly associated with IDH1 and indirectly interacted with IDH2 through IDH1. There were direct interactions among TP53, ATRX, IDH1, and IDH2 (**Figure 4A**). The combined score of the association showed that the highest score was that between IDH1 and IDH2 (0.976), followed by TP53 and ATRX (0.793), TP53 and IDH1 (0.770), IDH1 and ATRX (0.731), IDH2 and TP53 (0.700), ATRX and IDH2 (0.669), BRAF and TP53 (0.561), and BRAF and IDH1 (0.409) (**Figure 4B**).

## Discussion

Glioma is the most common primary brain malignancy and is characterized by high heterogeneity and extensive mutations (26). The roles of RAF serine/threonine protein kinases in various cancers have been investigated in the last two decades. *BRAF* regulates normal cell growth, differentiation, and survival *via* the MAPK/ERK pathway (27, 28). *BRAF* mutations and copy number variation have been widely investigated in melanoma, thyroid carcinoma, and lung and colon cancers (6, 29). Although *BRAF* ^V600E^ is rarely found in adult gliomas, it occurs predominately in pediatric gliomas, accounting for 68%–80% of pleomorphic xanthoastrocytoma (PXA), 20%–70% of ganglioglioma, 9%–10% of pilocytic astrocytoma (PA), 5%–15% of low-grade glioma (LGG), 20% of pediatric glioblastoma (pGBM), and 3% of adult glioblastoma multiforme (GBM) cases (30–32). Because genetic alterations are important in tumor development and progression (33, 34) and both *BRAF* ^V600E^ and *BRAF* ^AMP^ can activate the MAPK pathway, we investigated the different effects of these two *BRAF* alterations and the mutations associated on the survival of glioma patients.

In this study, among the various *BRAF* mutations that were identified using next-generation sequencing, the most frequent mutation was *BRAF* ^V600E^. Although some *BRAF* mutations are in the functional domains, other *BRAF* mutations with unknown functions occur across the gene (35). Patients with *IDH1* ^WT^ glioma have a poor prognosis; however, patients with *BRAF* ^V600E^ and *IDH1* ^WT^ experience favorable outcomes. Andrew S. Chi et al. report that five patients with grade II glioma harboring *BRAF* ^V600E^ without *IDH1* mutation who had undergone gross total resection without treatment were progression-free for 14–35 months; two patients with glioblastoma harboring *BRAF* ^V600E^ and *IDH1^WT^* had a progression-free survival of 36 and 19 months, respectively (36). In addition, a study reported a glioma patient with *BRAF* ^V600E^ without the *IDH1* mutation who experienced 2 years of overall survival (37). Hiromichi Suzuki’s study shows that *IDH* ^WT^ in grade II and III gliomas (type III) is associated with a poorer overall survival rate compared with that of glioblastoma. In contrast, the grade II subtype (type IIIa) was associated with more *BRAF* mutations and better overall survival than the grade III subtype (type IIIb) (26). Patients with glioma harboring *BRAF* ^V600E^ might benefit from MAPK pathway inhibitor target therapy, a rescue treatment that includes the use of RAF inhibitors and MEK inhibitors alone or in combination (38–41), and the results were encouraging (42). Our data show that the survival of the *BRAF* ^non-V600E^ cohort was comparable to that of the *BRAF* ^V600E^ cohort.

We also find that the gross survival of the *BRAF* ^AMP^ cohort was comparable to that of both the *BRAF* ^V600E^ and *BRAF* ^non-V600E^ cohorts. Because the *IDH1/2* mutation was frequently present in the *BRAF* ^AMP^ cohort, we divided this cohort into two groups based on the absence/presence of the *IDH1/2* mutation in order to elucidate the exact survival of patients with *BRAF* ^AMP^ alone and without the interference of the *IDH1/2* mutation. We found that the *BRAF* ^AMP^ and *IDH1/2* ^WT^ cohort had reduced overall survival compared with that of the *BRAF* mutation cohort (*BRAF* ^V600E^ and *BRAF* ^non-V600E^) and the *BRAF* ^AMP^ and *IDH1/2* ^MT^ groups. We propose two possible reasons for this. First, the mRNA and protein expression levels of *BRAF* ^AMP^ may be higher than those of *BRAF* ^V600E^, resulting in higher activation of the MAPK/ERK pathway and subsequent proliferation of cancer cells. Second, the survival of patients with *BRAF* ^AMP^ and *IDH1/2* ^MT^ was comparable to that of patients with *BRAF* mutations and greater than that of patients with *BRAF* ^AMP^ and *IDH1/2* ^WT^, probably because the *IDH1/2* mutation and 2-HG can induce oxidative stress, autophagy, and apoptosis in cancer cells. We believe that these two reasons may explain the poor survival of the *BRAF* ^AMP^ and *IDH1/2* ^WT^ cohorts. Young adult patients are enriched with *BRAF* ^V600E^ mutations and have better survival than older patients; we reveal that the survival of patients above 30 years of age in the *BRAF* ^AMP^ and *IDH1/2* ^WT^ cohort was also significantly reduced compared with that of the *BRAF* ^V600E^ cohort above 30 years (*P* = 0.018).

The results of *Cox* proportional hazard regression analysis show that *BRAF* ^AMP^ and *IDH1/2* ^WT^ genotype was an independent predictive factor for glioma with *BRAF* mutation and *BRAF* ^AMP^. *IDH1/2* mutations exist in greater than 70% of lower-grade gliomas (grades II and III) and in some glioblastomas (43, 44). It is known that the *IDH1/2* mutation leads to hypermethylation, which is the molecular basis of the CpG island methylator phenotype in gliomas (45). We found that *BRAF* ^AMP^ cohorts have lower survival compared with *BRAF* mutation cohorts, including *BRAF* ^V600E^ and *BRAF* ^non-V600E^. However, the survival of patients with *BRAF* ^AMP^ and *IDH1/2* ^MT^ was better than that of patients with *BRAF* ^AMP^ and *IDH1/2* ^WT^ and comparable to that of the *BRAF* ^non-V600E^ cohort. A previous study indicated that *IDH*1/2 mutation status alone was a predictive factor for longer overall survival and progression-free survival for the entire group of nonenhancing hemispheric grade II–III gliomas (46). Therefore, we propose that *IDH*1/2 mutations can improve the survival of cohorts with *BRAF* ^AMP^. Because the mutant *IDH1* and 2-HG can induce oxidative stress, autophagy, and apoptosis (47), we propose that this is the mechanism underlying the improvement in survival conferred by the *IDH*1/2 mutation.

Most of the studies of *BRAF* ^V600E^ in gliomas focus on pediatric neoplasms, especially in gangliogliomas and PXA (48–50). As all the patients enrolled in this study were adults, our findings provide insight into the effects of *BRAF* alterations in adult glioma. In addition to their diagnostic role, *BRAF* mutations may also have a prognostic value (51). Our data show that males accounted for the majority of patients in the *BRAF* mutation cohort, compared with the *BRAF* ^AMP^ cohort. The occurrence of GBM was higher in the *BRAF* mutation cohort than in the *BRAF* ^AMP^ cohort, whereas the *BRAF* ^AMP^ group had significantly more patients with the *IDH1/2*, *TP53*, and *ATRX* mutations. *ATRX* deletions/mutations are associated with several conventional molecular events, including *IDH1* and *TP53* mutations (52, 53). Somatic mutations in *TP53*, *ATRX*, and *IDH1/2* have been identified in adult low-grade gliomas (54). Although *IDH1/2* mutations are scarce in primary GBM, they are common in diffuse/anaplastic gliomas and secondary GBM (43, 44). *ATRX* mutations are detected in adult diffuse gliomas and astrocytomas harboring both *TP53* and *IDH1/2*. The co-occurrence of *TP53*, *IDH1/2*, and *ATRX* mutations facilitates the growth of a subgroup of adult diffuse astrocytomas (55). All of the above studies indicate that *ATRX* mutations frequently overlap with *IDH1* and *TP53* mutations. Additionally, our string analysis reveals close connections between *BRAF*, *IDH1*, *IDH2*, *TP53*, and *ATRX* proteins, similar to previous studies (55). Moreover, our results show that *BRAF* has direct reactions with *TP53* and *IDH1* but not with *ATRX*.

Active Ras can induce the hetero-dimerization of BRAF and CRAF (56), and BRAF can phosphorylate CRAF through direct protein–protein interactions (57, 58). CRAF exerts anti-apoptotic effects, which are mediated by an independent MAPK pathway (59, 60) through direct binding to Bcl-2 (59). TP53 can regulate Bcl-2 by suppressing Bcl-2 transcription (61). Liu et al. (55). find that ATRX alterations are correlated with mutations in *IDH1/2* and *TP53* in glioma of all grades. Lai et al. (62) find that the rate of Arg-to-Cys substitutions at position 273 in *TP53* is higher than that of Arg-to-His substitutions at position 132 in *IDH1*. They propose that this event is caused by a strand asymmetry mechanism (63) in which C to T mutations occur in the nontranscribed DNA strand in *TP53* and *IDH1* mutations occur in the transcribed strand in IDH. The study indicates that *IDH1/2* mutations represent early events in brain tumor formation (64). We propose that an increase in BRAF activates Bcl-2 by phosphorylating CRAF, and mutated *TP53* fails to regulate Bcl-2 but frequently accompanies *IDH1/2* mutation *via* a strand asymmetry mechanism. Further work using appropriate clinical tissue samples or animal models is required to provide some evidence for this proposal.

In conclusion, our study shows that *BRAF* ^AMP^ and *IDH1/2* ^WT^ is related to the reduced survival in adult patients with glioma compared with *BRAF* ^V600E^ and that *BRAF* ^AMP^ is associated with mutations in *IDH1*, *TP53*, and *ATRX*. This suggests that assessment for *BRAF* ^AMP^ and *IDH1/2^WT^* alterations is of prognostic value in adult glioma/glioblastoma patients because patients with this gene alteration pattern have relatively shorter survival times and may benefit from personalized, targeted therapy using *BRAF* and/or MEK inhibitors. As noted above, a concentrated effort is required to prospectively evaluate these findings in adult glioma patients.

## Data Availability Statement

The results published or shown here are in whole or part based upon data generated by the TCGA Research Network: https://www.cancer.gov/tcga.

## Ethics Statement

All data collected and generated from TCGA, which is a public database, and we strictly followed TCGA publication guidelines (https://www.cancer.gov/about-nci/organization/ccg/research/structural-genomics/tcga/using-tcga/citing-tcga).

## Author Contributions

RD, WW, MW, and HJ conceived and designed the work. RD and WW performed data analysis. RD, WW, and TW wrote the manuscript. MW and HJ revised the paper. All authors contributed to the article and approved the submitted version.

## Funding

This work was financially supported by the Natural Science Basic Research Program of Shaanxi (Program No. 2019JM-445, 2018JM7062) and The Project of The First Affiliated Hospital of Xi’an Jiaotong University (XJYFY-2019w33).

## Conflict of Interest

The authors declare that the research was conducted in the absence of any commercial or financial relationships that could be construed as a potential conflict of interest.

## Supplementary Material

Supplementary Figure 1 Kaplan-Meier Survival curves of patients with gliomas harboring *BRA*F^AMP^ and *BRAF* mutation. A: *BRAF* ^AMP^ cohort vs. *BRAF* mutation cohort (58.835 vs. 71.698, Chi-Square 0.020, P = 0.886);B: *BRAF* ^V600E^ cohort vs. *BRAF* ^non-V600E^ cohort (41.573 vs. 89.958, Chi-Square 1.999, P = 0.157);C: *BRAF* ^AMP^ & *IDH1/2* ^MT^ cohort vs. *BRAF* ^V600E^ cohort (67.026 vs. 41.573, Chi-Square 1.031, P = 0.310);D: *BRAF* ^AMP^ & *IDH1/2* ^MT^ cohort vs. *BRAF* ^non-V600E^ cohort (67.026 vs. 89.958, Chi-Square 0.025, P = 0.875);E: *BRAF* ^AMP^ & *IDH1/2* ^MT^ cohort vs. *BRAF* mutation cohort (67.026 vs.71.698, Chi-Square 0.513, P = 0.474). BRAF, v-raf murine viral oncogene homolog B1; IDH1/2, isocitrate dehydrogenase 1 and 2.

Supplementary Figure 2 Kaplan-Meier Survival curves of patients above 30 years of age with gliomas harboring *BRAF* ^AMP^& *IDH1/2* ^WT^ and *BRAF* ^V600E^. *BRAF* ^AMP^ & *IDH1/2* ^WT^ cohort vs. *BRAF* ^V600E^ cohort (9.750 vs. 40.135, chi-square 5.575, P = 0.018). BRAF, v-raf murine viral oncogene homolog B1; IDH1/2, isocitrate dehydrogenase 1 and 2.
