# Supplementary figures and images for: BRAF AMP Frequently Co-occurs With IDH1/2, TP53, and ATRX Mutations in Adult Patients With Gliomas and Is Associated With Poorer Survival Than That of Patients Harboring BRAF V600E
Source: Front Oncol. 2021 Jan 7;10:531968. doi: 10.3389/fonc.2020.531968 (PMC7817544; doi:10.3389/fonc.2020.531968)

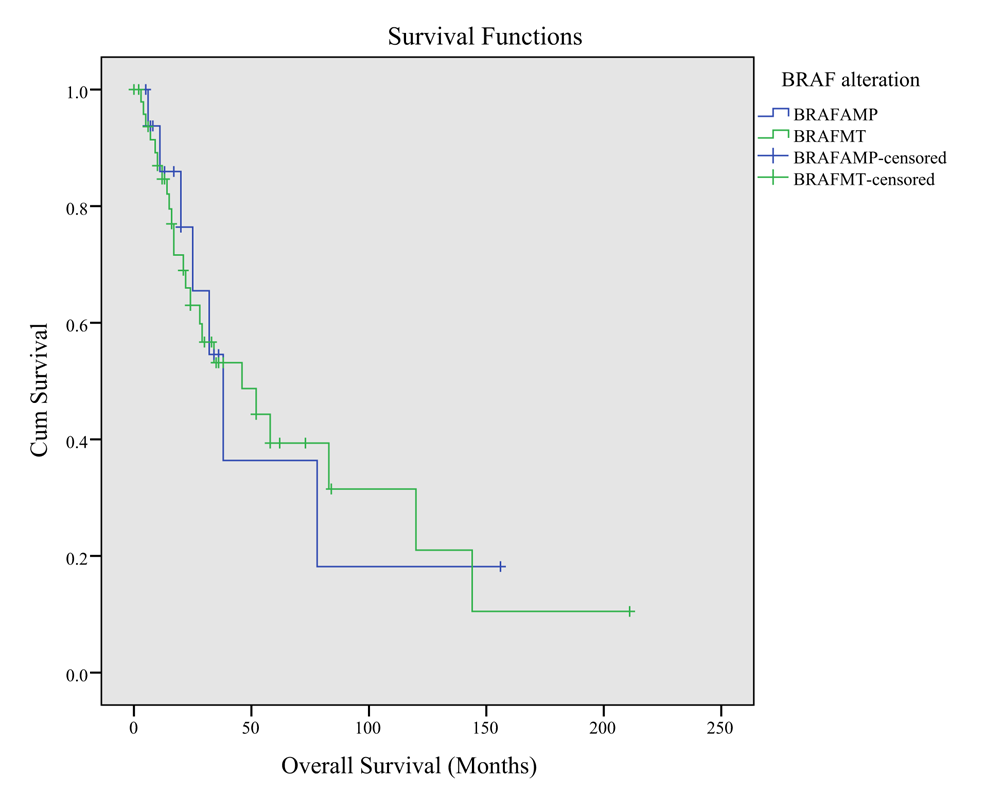

Supplement: Supplementary Figure 1 — Kaplan-Meier Survival curves of patients with gliomas harboring BRAFAMP and BRAF mutation. A: BRAF AMP cohort vs. BRAF mutation cohort (58.835 vs. 71.698, Chi-Square 0.020, P = 0.886);B: BRAF V600E cohort vs. BRAF non-V600E cohort (41.573 vs. 89.958, Chi-Square 1.999, P = 0.157);C: BRAF AMP & IDH1/2 MT cohort vs. BRAF V600E cohort (67.026 vs. 41.573, Chi-Square 1.031, P = 0.310);D: BRAF AMP & IDH1/2 MT cohort vs. BRAF non-V600E cohort (67.026 vs. 89.958, Chi-Square 0.025, P = 0.875);E: BRAF AMP & IDH1/2 MT cohort vs. BRAF mutation cohort (67.026 vs.71.698, Chi-Square 0.513, P = 0.474). BRAF, v-raf murine viral oncogene homolog B1; IDH1/2, isocitrate dehydrogenase 1 and 2. [file Image_1.tif]

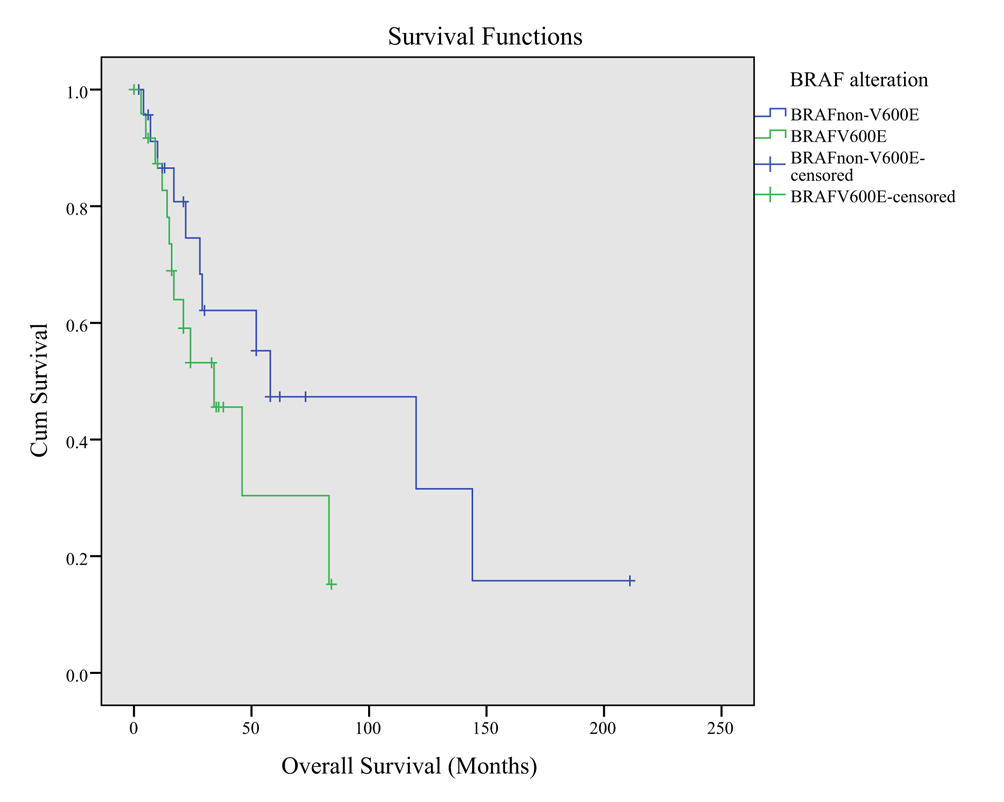

Supplement: Supplementary Figure 2 — Kaplan-Meier Survival curves of patients above 30 years of age with gliomas harboring BRAF AMP& IDH1/2 WT and BRAF V600E. BRAF AMP & IDH1/2 WT cohort vs. BRAF V600E cohort (9.750 vs. 40.135, chi-square 5.575, P = 0.018). BRAF, v-raf murine viral oncogene homolog B1; IDH1/2, isocitrate dehydrogenase 1 and 2. [file Image_2.tif]

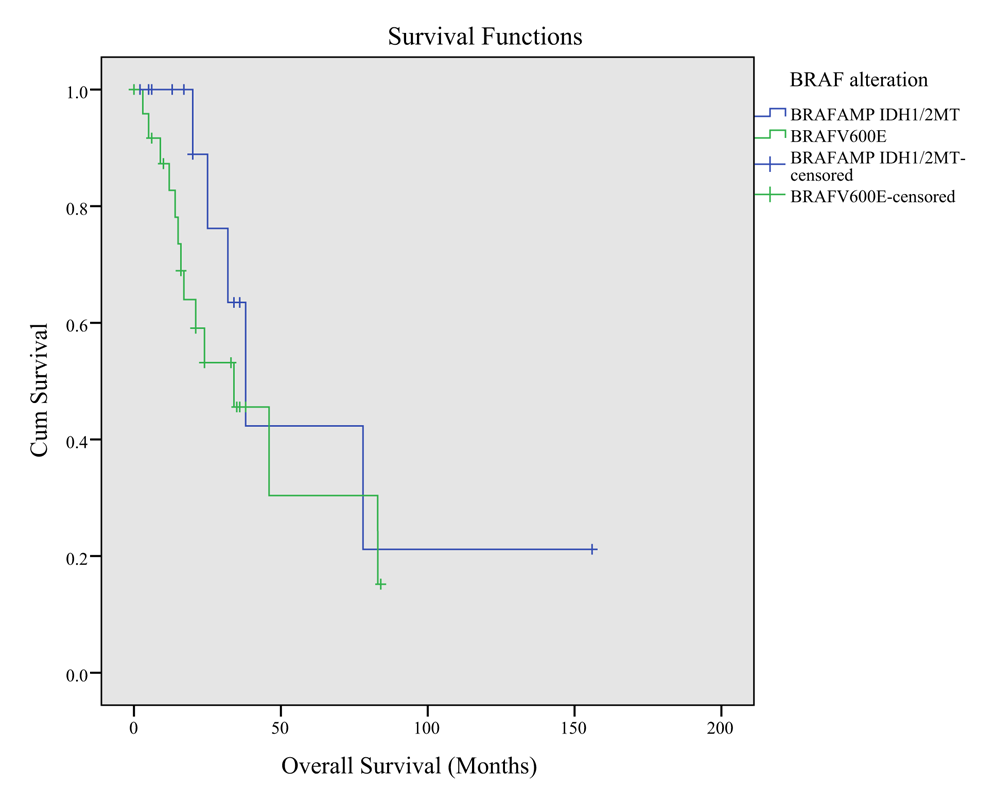

Supplement: Supplementary file 3 [file Image_3.tif]

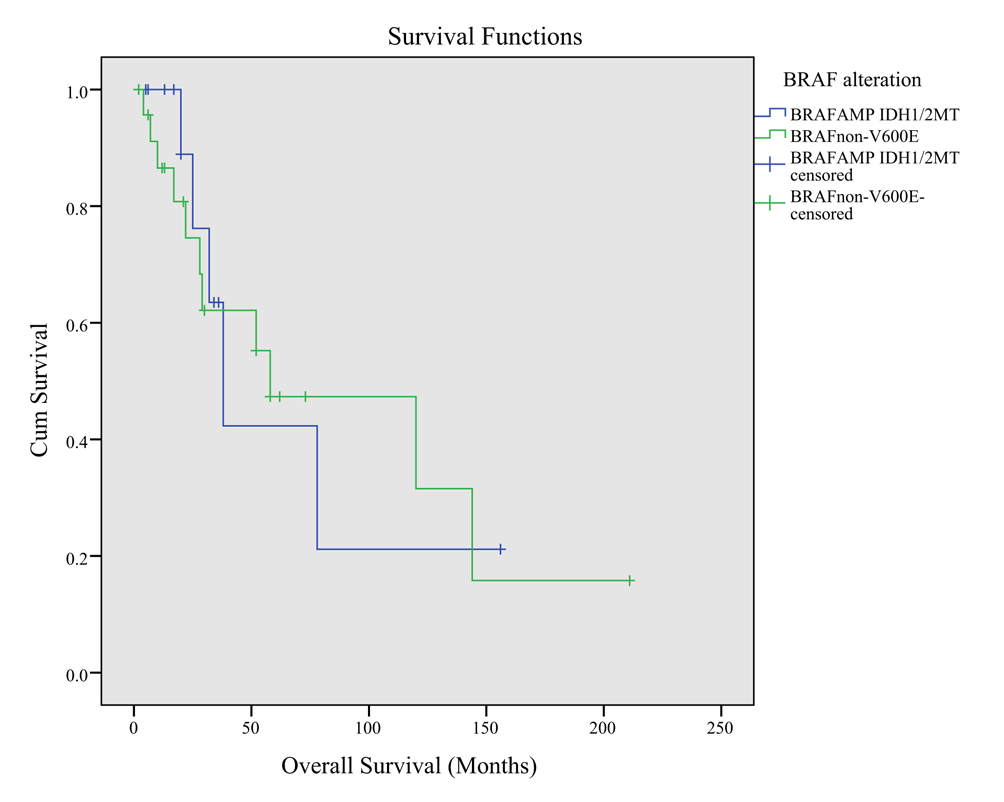

Supplement: Supplementary file 4 [file Image_4.tif]

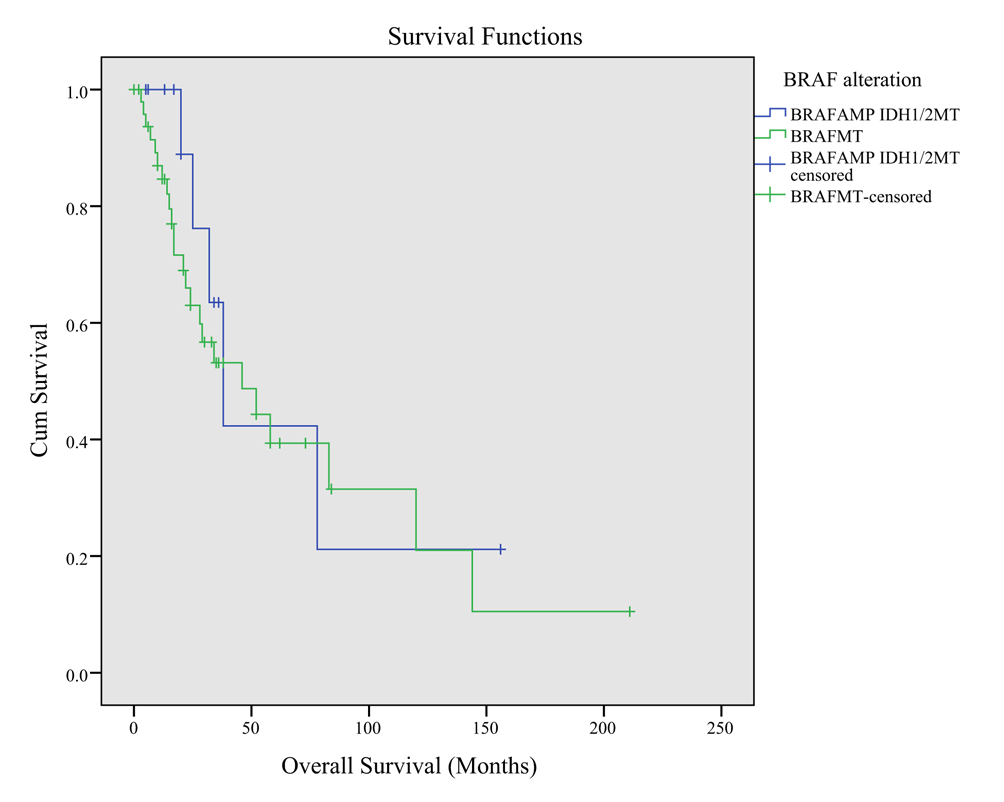

Supplement: Supplementary file 5 [file Image_5.tif]

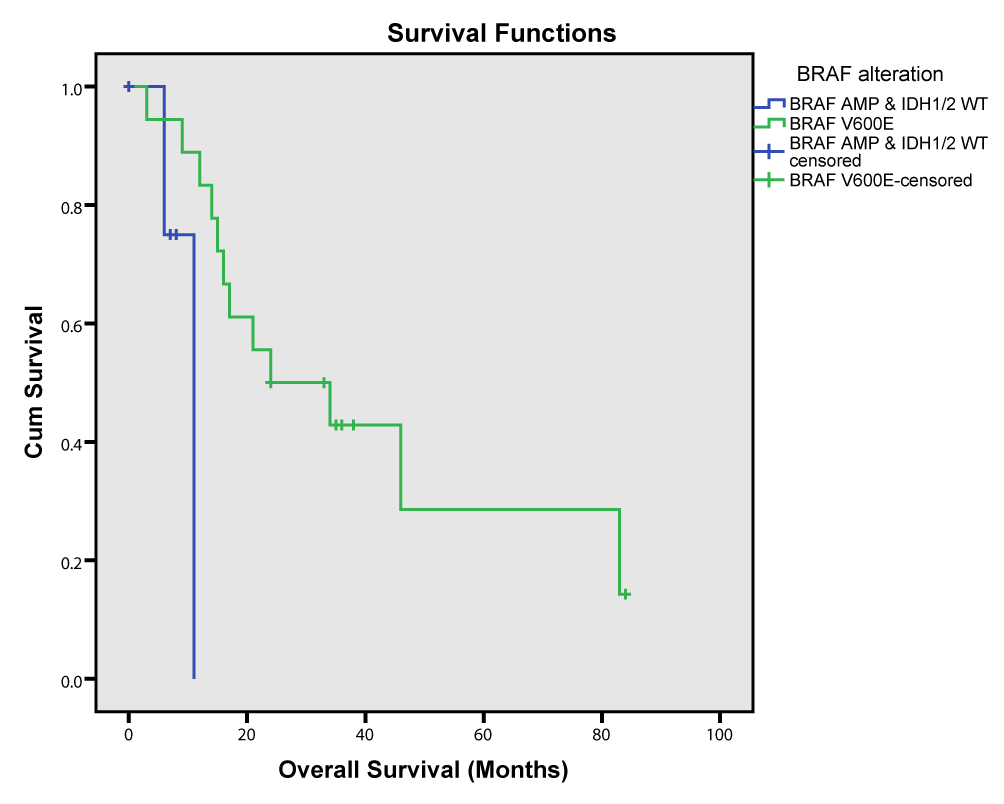

Supplement: Supplementary file 6 [file Image_6.tif]
